# Changes in the body mass index and blood pressure association across time: Evidence from multiple cross-sectional and cohort studies

**DOI:** 10.1016/j.ypmed.2021.106825

**Published:** 2021-12

**Authors:** David Bann, Shaun Scholes, Rebecca Hardy, Dara O'Neill

**Affiliations:** a Centre for Longitudinal Studies, Social Research Institute, University College London, London, UK; b Department of Epidemiology and Public Health, University College London, London, UK; c CLOSER, Social Research Institute, University College London, London, UK

**Keywords:** Body mass index, Obesity, Blood pressure, Hypertension, Trends

## Abstract

Although body mass index (BMI) is considered a key determinant of high blood pressure, its importance may differ over time and by age group. We utilised separate data sources to investigate temporal changes in this association: 23 independent (newly sampled), repeated cross-sectional studies (Health Survey for England (HSE)) at ≥25 years (1994–2018; *N* = 126,742); and three British birth cohorts at 43–46 years (born 1946, 1958, and 1970; *N* = 18,657). In HSE, associations were weaker in more recent years, with this trend most pronounced amongst older adults. After adjustment for sex, anti-hypertensive treatment and education, the mean difference in systolic blood pressure (SBP) per 1 kg/m^2^ increase in BMI amongst adults ≥55 years was 0.75 mmHg (95%CI: 0.60–0.90) in 1994, 0.66 mmHg (0.46–0.85) in 2003, and 0.53 mmHg (0.35–0.71) in 2018. In the 1958 and 1970 cohorts, BMI and SBP associations were of similar magnitude yet weaker in the 1946 cohort, potentially due to differences in blood pressure measurement device. Quantile regression analyses suggested that associations between BMI and SBP were present both below and above the hypertension threshold. A weaker association between BMI and blood pressure may partly offset the public health impacts of increasing obesity prevalence. However, despite sizable increases in use of antihypertensive medication, BMI remains positively associated with SBP in all ages. Our findings highlight the need to tackle non-medical factors such as population diet which influence both BMI and blood pressure, and the utility of using multiple datasets to obtain robust inferences on trends in risk factor-outcome associations across time.

## Highlights

• We investigated trends in body mass index (BMI) and blood pressure (BP) associations from 1989 to 2018.
• In analysis of 23 cross-sectional studies, BMI and BP associations weakened, particularly in older adults.
• Evidence from 3 birth cohorts was inconsistent and potentially biased by measurement differences.
• Weakening links between BMI and outcomes may partly offset the impact of the obesity epidemic.
• Results highlight the utility of using multiple datasets to obtain robust inferences on trends across time.

## 1 Introduction

High body mass index (BMI) is an important modifiable determinant of high blood pressure, as evidenced by meta-analyses of observational studies (Jayedi et al., 2018) and weight-loss interventions (Neter et al., 2003). While providing precise estimates of association between BMI and blood pressure, such studies have not elucidated whether this association has changed across time. Evidence from British birth cohorts born in 1946 and 1958 suggested an approximate doubling of the positive correlation between BMI and blood pressure in midlife from 1989 to 2003 (Li et al., 2015). Other studies have reported increasing strength of association, yet used hypertension as the sole outcome (Nagai et al., 2015; Ryu et al., 2019) (defined as elevated blood pressure or use of antihypertensive medication) and thus may be simply reflecting increases in treatment use (Tanamas et al., 2017). In contrast, other studies have reported weakening of the association (Danon-Hersch et al., 2007; Adler et al., 2015), or reported no clear systematic change (Tanamas et al., 2017).

Understanding whether the associations between BMI and blood pressure have changed across time is important to inform future public health responses to obesity, the prevalence of which has increased markedly since the 1980s (Ng et al., 2014; Finucane et al., 2012; NCD Risk Factor Collaboration, 2016) and may continue to increase (Wang et al., 2011). If the risks of rising BMI on raised blood pressure levels are also increasing, then the public health impacts of BMI may be greater than anticipated given the importance of high blood pressure for a range of cardiovascular (Bundy et al., 2017) and neurological outcomes (Sharp et al., 2011; Shah et al., 2009).

Multiple factors could explain why associations between BMI and blood pressure may differ across time (Critchley and Cooper, 2018). Recent decades have seen improvements in hypertension awareness, detection, and treatment—notably via increased use of antihypertensive medication (Zhou et al., 2019). Such interventions, along with behavioural changes triggered at least in part by hypertension diagnosis in primary care (National Institute for Health and Care Excellence, 2019), may weaken such associations by lowering raised blood pressure levels amongst those at highest cardiovascular risk, including those with higher BMI. However, treatment only applies to the minority (<25%; Zhou et al., 2017) of the population above specified treatment thresholds and even if initiated it may not fully normalise blood pressure levels (Tobin et al., 2005; Balakrishnan et al., 2017). Further, obtaining long-term changes to risk factors such as diet and physical activity is notoriously challenging. Other changes which may also impact on the strength of BMI and blood pressure associations include secular changes to body composition. A high BMI may reflect more fat mass in more recent decades (Nagel et al., 2009; Sun et al., 2012), and since high fat rather than high muscle mass is thought to impact on high blood pressure (Neter et al., 2003), associations may in turn have strengthened across time.

We investigated whether BMI and blood pressure associations systematically changed across time from 1989 to 2018, employing data from two separate sources: British birth cohort studies and independent (newly-sampled), repeated cross-sectional English studies. Each has complementary advantages. The birth cohorts have comparable blood pressure measures obtained in midlife—a purportedly important period for cardiovascular risk (Gottesman et al., 2017; Lane et al., 2019; Wills et al., 2011)—and early life measures of potential confounding factors. Cross-sectional studies in turn collect data more regularly and generalise to a broader age span.

## 2 Methods

We used two data sources: 1) cross-sectional studies (Health Survey for England; HSE), with anthropometrics and blood pressure measured in 23 independent samples of adults (undertaken annually from 1994 to 2018, except 1999 and 2004 where blood pressure was measured only amongst minority ethnic groups); and 2) three British birth cohort studies focussed on health and social characteristics of persons born in 1946 (1946c), 1958 (1958c) and 1970 (1970c), with anthropometrics and blood pressure measured in midlife (in 1989, 2003, and 2016; see cohort profiles (Wadsworth et al., 2006; Power and Elliott, 2006; Connelly and Platt, 2014)) (Bann et al., 2020). The purpose of the HSE is to monitor the health of the population, and to feed the results of any analyses into the development of health policy, strategy and management (Mindell et al., 2012); birth cohort studies are multidisciplinary longitudinal studies with prospective data on health and social factors across life.

For each, participants gave verbal and/or written consent to be interviewed, visited by a nurse, and to have weight, height, and blood pressure measurements taken during a home visit (Bann et al., 2020). BMI was calculated as weight (kg) divided by the square of height (m^2^). Briefly, each study used standardised protocols after 5 min rest periods. Omron devices were used in 1958c (705CP) and 1970c (HEM-907), and in HSE from 2003 onwards. 1946c used the Hawksley random zero sphygmomanometer and HSE before 2003 used the Dinamap 8100 monitor. The 1946c and pre-2003 HSE data were converted to Omron measures using previously published regression equations based on calibration studies (Stang et al., 2006; Falaschetti et al., 2009). Research ethics approval was obtained from relevant committees and are on file with the authors' institutions; all data used were anonymised.

The analytical sample size was 145,399—those with outcomes at age 43–46 years (cohorts; *N* = 18,657) or 25 years and over (HSE; *N* = 136,942 with blood pressure and educational data; *N* = 126,742 with blood pressure, BMI and educational data); for flowcharts see Supplementary Figs. 1–2. The lower age limit of 25 years was chosen for HSE to enable measurement of highest educational attainment (a key confounding variable) for the majority of participants after completing continuous full-time education. The primary outcome for our study was the continuous level of systolic blood pressure (SBP), chosen a priori given its strong link with cardiovascular disease and mortality (Bundy et al., 2017); diastolic blood pressure (DBP) was used as a secondary outcome.

## 3 Analytical strategy

### 3.1 Accounting for treatment use

Our main analyses accounted for treatment use since changes in this variable could confound time-trend differences in BMI and blood pressure associations (Tanamas et al., 2017). We accounted for the expected average effects of antihypertensive medication use on blood pressure by adding a constant of 10 mmHg (SBP) and 5 mmHg (DBP) to the observed (“raw”) values amongst those on treatment. This adjustment method approximates “underlying” SBP/DBP (i.e. the SBP/DBP individuals would have if they were not on treatment) (Law et al., 2003); this method has been found to reduce bias in the estimated effect of key determinants on continuous blood pressure levels (Tobin et al., 2005; Balakrishnan et al., 2017).

### 3.2 Associations between BMI and SBP

Birth cohort and HSE datasets were analysed separately using linear regression. Data from each source was analysed overall (i.e., pooled across cohorts/survey years) and also separately by birth cohort/survey year. We plotted histograms of BMI and SBP/DBP, and used linear regression to estimate mean difference in SBP per unit increase in BMI. Analyses were first adjusted for sex and then education attainment—a potential confounder (common cause of both BMI and blood pressure levels (Bann et al., 2020; Davies et al., 2018)), included as a categorical term comprising four groups: degree/higher (reference), A levels/diploma, O Levels/GCSEs/vocational equivalent, or none. In cohort studies, additional adjustment was made for social class at birth, maternal education in childhood, and own social class at 43-46y. HSE models additionally adjusted for age. These analyses were repeated with DBP as the outcome.

### 3.3 Associations between BMI and SBP by HSE survey year and by age group

A formal test of change across time in the BMI-SBP association was examined in the pooled HSE dataset by fitting interaction terms (a negative sign indicated a weakening association in subsequent years). Trend analysis was conducted separately in two time-periods (1994–2002 and 2003–2018) to examine the influence of change in blood pressure measurement device and introduction of non-response weights.

Given age differences in body composition (Jackson et al., 2012) and treatment use (Zhou et al., 2019), the BMI-SBP association may differ by age. We therefore conducted additional analyses in younger (25–54 years; *N* = 73,750) and older age groups (55+ years; *N* = 52,992). HSE analyses were also restricted to 40–49 year-olds (*N* = 25,941) to compare with cohort data.

### 3.4 Sex-stratified analyses (HSE and cohorts)

We additionally conducted sex-stratified analyses to investigate if the time-trends in BMI and blood pressure associations differed by sex.

### 3.5 Quantile regression

Quantile regression (Koenker and Bassett, 1978) on data pooled across cohorts/survey years (HSE) was used to estimate whether the observed mean difference in SBP per unit increase in BMI were driven by differences at the upper or lower part of the SBP distribution; such analyses help inform the possible impact of treatment use on BMI and SBP associations. Estimates were obtained and plotted at the 5th, 10th, 25th, 50th (median), 75th, 90th, and 95th quantiles. To facilitate interpretation, we plotted the quantiles corresponding to the 140 mmHg SBP threshold for initiating blood pressure lowering treatment (National Institute for Health and Care Excellence, 2019).

### 3.6 Missing data

To address data missingness, multiple imputation by chained equations was completed, with 20 imputations performed for the cohort pooled data and 20 each for the sex-stratified cohort datasets (including all socioeconomic position indicators and BMI). The blood pressure outcome was included in the imputation modelling but not imputed. Weighting was used in lieu of multiple imputation in HSE analyses. Starting from 2003, weights have been created to minimise non-response bias (including non-participation in the nurse visit amongst those interviewed at the first stage); the relevant weights for analysing blood pressure data were therefore used from 2003 onwards.

## 4 Results

Table 1 presents descriptive statistics for participants' characteristics. In later (2016/18) compared with earlier years (1989–2004), mean BMI and obesity prevalence (BMI ≥30 kg/m^2^) were higher (Table 1). In HSE but not cohort datasets, mean SBP and hypertension prevalence (140/90 mmHg or use of antihypertensive medication) were lower in later years. Reported use of blood pressure lowering medication was more frequent in later years in both data sources (Table 1). The distributions of BMI were wider and more right-skewed in later compared with earlier years, while SBP was more normally distributed in each year (Supplementary Fig. 3).

**Table 1:** Participant characteristics: data from 23 independent, repeated cross-sectional English studies and three British birth cohort studies.

	Repeated cross-sectional study (≥25y), year of outcome measurement			Birth cohort study in midlife (43-46y), year of outcome measurement (birth year)		
	1994	2003	2018	1989 (1946)	2003 (1958)	2016 (1970)
Sample size	11,427	7910	3748	3186	8610	6861
25–54	7122	4487	1771	–	–	–
55+	4305	3423	1977	–	–	–
Outcomes						
Systolic blood pressure, mean (SD) mmHg*	133.6 (18.3)	129.9 (19.1)	125.3 (16.8)	125.9 (15.5)	126.4 (17.0)	124.1 (15.5)
Diastolic blood pressure, mean (SD) mmHg*	75.1 (8.9)	74.9 (11.5)	73.4 (11.2)	81.6 (10.4)	78.8 (11.1)	77.1 (11.2)
Hypertension, %**	35.5%	33.8%	31.6%	23.8%	28.4%	24.1%
Blood-pressure lowering medication, %	13.5%	14.3%	18.6%	3.8%	6.1%	7.5%
Blood-pressure lowering medication, N missing	–	–	–	41	0	1
Adjusted systolic blood pressure, mean (SD) mmHg***	134.9 (19.5)	131.3 (20.4)	127.1 (18.3)	126.3 (15.8)	127.0 (17.4)	124.9 (16.1)
Adjusted diastolic blood pressure, mean (SD) mmHg***	75.8 (9.3)	75.6 (11.7)	74.3 (11.4)	81.8 (10.5)	79.1 (11.3)	77.5 (11.5)
Exposure						
Body mass index, mean (SD) kg/m^2^	26.2 (4.3)	27.4 (4.9)	27.9 (5.6)	25.6 (4.2)	27.4 (4.9)	28.4 (5.5)
Body mass index, N missing	402	592	376	11	117	256
Obesity, %	16.4%	24.5%	29.7%	12.6%	44.7%	46.9%
Socioeconomic data						
Social class at birth-4y, % manual	–	–	–	74.4%	70.2%	64.7%
Social class at birth-4y, N missing	–	–	–	187	109	147
Own education attainment, % degree	11.1%	19.3%	32.7%	6.6%	9.2%	27.3%
Own education attainment, N missing****	226	424	44	174	885	1197

Notes: With valid blood pressure data. 2003 selected in HSE as approximate mid-point in year range to facilitate comparison with cohort data (same year as 1958c observation) and because of its relatively large sample size, change in BP measurement device and the introduction of non-response weighting. N: count. SD: standard deviation. * Raw SBP/DBP values (i.e., not adjusted for use of antihypertensive medication); HSE sample size is unweighted, estimates are weighted (2003 and 2018). **Hypertension: SBP/DBP ≥ 140/90 mmHg or reported use of antihypertensive medication. ***SBP/DBP values adjusted for use of antihypertensive medication (by adding a constant of 10 mmHg to SBP and 5 mmHg to DBP for those using antihypertensive medication). Estimates for SBP/DBP include participants with missing BMI.^⁎⁎⁎⁎^ Missing educational data includes those with ‘other’ qualifications.

### 4.1 Associations between BMI and SBP

**Fig. 1:**
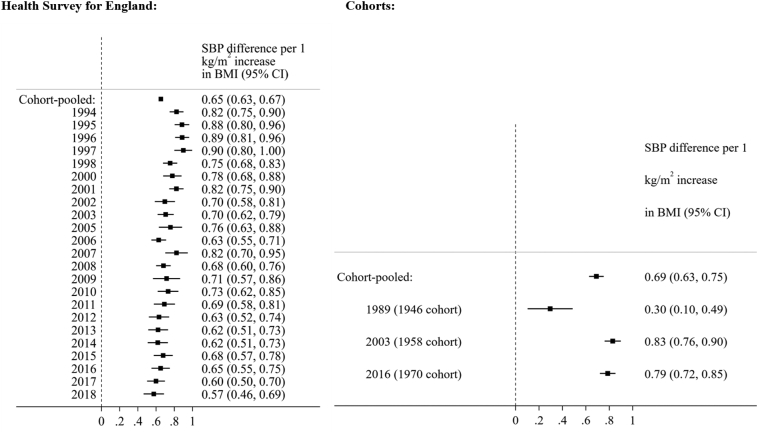
Associations between body mass index (kg/m^2^) and systolic blood pressure (mmHg) across adulthood (≥25 years, from independent, repeated cross-sectional English data, left panel; *N* = 126,742), and midlife (43–46 years, from British birth cohort data, right panel; *N* = 18,657). Note: raw SBP values adjusted for use of antihypertensive medication (by adding a constant of 10 mmHg for those using antihypertensive medication). HSE models are adjusted for sex, age, and education. Cohort models are also adjusted for mother's education and cohort member's own education, social class at birth, and social class in midlife. Sample sizes in each cohort / survey year shown in Supplementary Figs. 1 and 2, respectively.

BMI was positively associated with SBP in all HSE survey years and cohorts (Fig. 1). The trends in this association (by survey year/cohort) were similar before and after accounting for treatment, education attainment, and (in cohorts) multiple socioeconomic indicators across life (Supplementary Fig. 4).

### 4.2 Associations between BMI and SBP by HSE survey year and by age group

In HSE across all ages (≥25 years), BMI-SBP associations were weaker in subsequent years (Fig. 1; BMI*year interaction term: β = −0.011 (95% CI: −0.014, −0.008)). Across all time points there was considerable year-to-year variability in the magnitude of association (Fig. 1).

Trend analysis conducted separately in 1994–2002 and 2003–2018 (when HSE changed blood pressure measurement device and non-response weights were introduced) yielded similar findings (Supplementary Table 1).

**Fig. 2:**
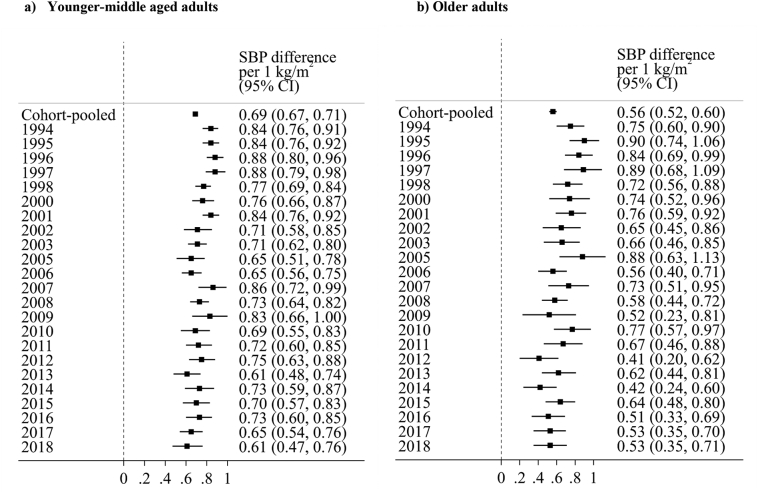
Associations between body mass index (kg/m^2^) and systolic blood pressure (mmHg) in 25–54 years (left panel) and 55+ years (right panel, from independent, repeated cross-sectional English data; *N* = 126,742). Note: SBP values adjusted for use of antihypertensive medication (by adding a constant of 10 mmHg for those using antihypertensive medication). Models are also adjusted for sex, age, and education. Sample sizes in each survey year are shown in Supplementary Fig. 2.

In age-stratified linear regression analyses, the BMI-SBP association was stronger in younger (25–54 years) compared with older (≥55 years) adults (Fig. 2) as shown by the non-overlapping 95% CIs for data pooled across survey years (25–54 years: β = 0.69 (95% CI: 0.67–0.71); ≥55 years: β = 0.56 (95% CI: 0.52–0.60)). However, a weakening of association across time was more pronounced amongst older adults—mean differences in SBP per 1 kg/m^2^ increase in BMI (after accounting for treatment and education) were 0.75 mmHg (0.60, 0.90) in 1994, 0.66 mmHg (0.46, 0.85) in 2003, and 0.53 mmHg (0.35, 0.71) in 2018. Owing to the lower sample sizes in the older age group, confidence intervals were wider. Consistent with this interpretation, evidence for a weakening association in subsequent years was stronger in older (BMI*year interaction term: β = −0.010 (−0.015, −0.005)) compared with younger adults (β = −0.006 (−0.009, −0.003)), yet confidence intervals overlapped (Supplementary Table 1).

In contrast to older adults (aged ≥55 years), our analyses restricted to 40–49 year-olds (to compare more specifically with cohort data) showed no evidence of a weakening BMI-SBP association in subsequent years (Supplementary Fig. 5 and Supplementary Table 1; BMI*year interaction term: β = −0.002 (−0.007, 0.003)).

### 4.3 Associations between BMI and SBP by birth cohort

In cohorts (SBP measures at 43-46y only), the magnitude of association was substantially smaller in 1946c; magnitudes were highest in 1958c and slightly weaker in 1970c—the mean difference in SBP per 1 kg/m^2^ increase in BMI (after accounting for treatment and all socioeconomic indicators) was 0.30 mmHg (0.10, 0.49) in 1946c, 0.83 mmHg (0.76, 0.90) in 1958c, and 0.79 mmHg (0.72, 0.85) in 1970c (Fig. 1). Highly comparable estimates were observed when cohort data was restricted to English residents only (Supplementary Fig. 6).

### 4.4 Quantile regression analyses

**Fig. 3:**
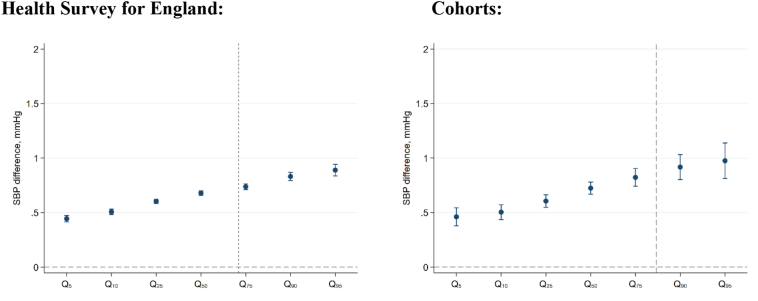
Associations between body mass index and systolic blood pressure quantiles (mmHg) across adulthood (≥25 years, from repeated cross-sectional data, left panel; N = 126,742), and midlife (43–46 years, from birth cohort data, right panel; *N* = 18,657). Estimates show the difference in SBP per 1 unit increase in BMI at specified centiles of the SBP distribution; for example, Q50 shows the difference at the median. Note: raw SBP values adjusted for use of antihypertensive medication (by adding a constant of 10 mmHg for those using antihypertensive medication); estimates are adjusted for sex and education attainment.

Quantile regression results for data pooled across cohorts/survey years suggested that BMI-SBP associations were present across the SBP distribution—below and above the treatment threshold—yet were larger at upper SBP values (Fig. 3 shows findings after accounting for treatment—findings were similar using “raw” blood pressure values). For example, in cohort-pooled analyses there was a 0.72 mmHg (0.67–0.78) difference in SBP at the 50th quantile (median) per 1 kg/m^2^ increase in BMI, yet a 0.92 mmHg (0.80–1.03) difference in SBP at the 90th quantile. Comparable findings were found in HSE (Fig. 3), and in general, this pattern of results was similar in each survey year (Supplementary Fig. 7).

### 4.5 Associations between BMI and DBP

In HSE, the magnitude of BMI-DBP associations were weaker prior to 2003, highest in 2003, and weaker again in 2018 (Supplementary Fig. 8). In cohort analyses, results using DBP yielded comparable results to those for SBP: BMI-DBP associations were similarly weaker in 1946c, and higher in 1958c and 1970c (also Supplementary Fig. 8).

### 4.6 Sex-stratified analyses

Overall, trends in the association between BMI and both SBP and DBP were largely similar in men and women in HSE and cohort datasets (Supplementary Figs. 9–10).

## 5 Discussion

Using data from 23 independent, repeated cross-sectional English datasets, we found that associations between BMI and SBP were weaker from 1994 to 2018; this trend was strongest in older (≥55 years) compared with younger (25–54 years) adults. Analysis of three British birth cohorts with data in midlife (43–46 years) did not provide clear evidence of systematic trend in the association; in repeated cross-sectional data, there was weaker evidence for systematic change in the equivalent age group (40–49 years). However, BMI remained positively associated with SBP in all age groups, highlighting the future adverse consequences of the ongoing obesity epidemic.

The discrepancy in findings between HSE and the birth cohorts requires explanation. The fact that the increasing strength of the cross-sectional BMI-SBP associations previously reported (from 1946c to 1970c—i.e., 1989–2000) (Li et al., 2015) were not found in 1970c, nor in the 23 HSE datasets used (1994–2018), suggests that this finding may be attributable to particular features of the 1946c rather than reflecting a long-term trend. This may include chance differences in findings due to sampling. The 1946c was sampled in one week of March 1946, and only married mothers were included. Unobserved differences in BMI and blood pressure measurement protocols could have also contributed to such differences. More measurement error in calculating BMI would lead to a weakening of BMI-SBP associations (due to regression dilution bias (Hutcheon et al., 2010)), yet is an unlikely explanation since similar protocols for BMI measurement were used in each cohort. Notably, the blood pressure measurement devices used differed in each cohort. While the weaker BMI-SBP association in 1946c was observed both before and after applying calibration equations to account for device differences (data available on request), it is possible that the calibration did not calibrate SBP measures equivalently. Indeed, the mean difference in SBP per 1 kg/m^2^ increase in BMI at a later age in 1946c (53y, when an Omron device was used) was substantially larger than at 43y (β(at 53y) =1.09 mmHg (0.91–1.27) after accounting for treatment, vs 0.34 mmHg (0.14–0.53) at 43y). Such discrepancies may also explain the finding that BMI-DBP associations were weaker in HSE prior to 2003, coinciding with a similar change in device. If the older devices are responsible for these weaker associations with BMI in the 1946c and in HSE 1994–2002, it suggests that differential error has occurred (e.g., systematic underestimation of SBP/DBP amongst those with higher BMI, or overestimation amongst those with lower BMI). Datasets with SBP measures obtained using multiple devices and measured anthropometrics are required to test this possibility—it has broader implications given the possibility that it may bias comparisons made within (longitudinally) and between studies.

Our study extends and potentially clarifies the equivocal existing literature which examined trends across time in cross-sectional BMI-blood pressure associations (Li et al., 2015; Nagai et al., 2015; Ryu et al., 2019; Tanamas et al., 2017; Danon-Hersch et al., 2007; Adler et al., 2015; Zhang et al., 2020; Tu et al., 2011). We used two different sets of studies with complementary features over a long time-span. Considerable year-to-year variability was observed in the magnitude of these associations, illustrating how comparisons restricted to two survey years may lead to misleading conclusions regarding long-term trends. Two previous studies (in the United States and Japan) in which an increasing strength of association was reported (Nagai et al., 2015; Ryu et al., 2019; Zhang et al., 2020) used hypertension as an outcome (i.e., high blood pressure or use of blood pressure lowering treatment), and their findings may thus be solely explained by the secular trend of rising treatment use (increasing hypertension prevalence disproportionately amongst those at highest cardiovascular risk) rather than changes in the BMI and blood pressure association per se (Tanamas et al., 2017).

In studies that used continuous BMI and blood pressure measures, weakening of the BMI and blood pressure association across time has also been observed in Germany (1998–2011) (Adler et al., 2015), the Seychelles (1989 vs 2004) (Danon-Hersch et al., 2007), and the US (1993–2007 and 2005–2012, in some but not all model specifications) (Tanamas et al., 2017). Another in contrast reported a strengthening relationship in Taiwan (1998–2006) (Tu et al., 2011), yet this study was restricted to a ‘healthy sample’ aged 20–59 years who were free of common chronic diseases and who were not on long-term medication (including antihypertensives). We further extend the existing literature by utilising quantile regression analysis—findings of which suggest that the influence of BMI on SBP appears to be present across the SBP distribution (below and above the hypertension treatment threshold).

The magnitude of BMI and underlying blood pressure associations is likely modified both by factors which could weaken the association across time (such as reduced salt intake amongst those with higher BMI) and those which strengthen it (such as a higher fat mass for a given BMI value in more recent decades). While we lack time series data on these factors in the studies utilised, our findings suggesting persistent associations from 2003 to 2018 amongst younger-middle aged adults implies that these differing processes may have offset each other, leading to similar magnitudes of association. In older adults, the relative balance of these factors seemingly led to a weakening of association across time. While we lack direct evidence for increases in fat mass percent in this study context, evidence from the US and elsewhere does suggest that it may have occurred along with the increasing obesity epidemic (Nagel et al., 2009; Sun et al., 2012). Recent public health initiatives in the United Kingdom have included screening programmes for higher blood pressure levels amongst those at highest cardiovascular disease risk (Serumaga et al., 2011; Chang et al., 2019), leading to increases in hypertension diagnosis and treatment amongst those with high BMI (Scholes et al., 2021). While treatment has increased, as observed in our data, it has seemingly had a modest effect on BMI and blood pressure associations, particularly in younger age groups. This is evidenced by 1) our analysis accounting for treatment, and 2) quantile regression analyses suggesting that much of the BMI-SBP association is present below the 140 mmHg threshold for antihypertensive treatment. Changes in health behaviours may also have played a role in these processes. Salt intake has declined in the United Kingdom in recent decades (He et al., 2014), and this may have been particularly beneficial to those with higher BMI. Further weakening of the link between BMI and blood pressure could feasibly be achieved by additional improvements to the risk factor profile amongst those with higher BMI—such as reduced salt and calorie intake, and increased physical activity.

### 5.1 Strengths and limitations

Strengths include the use of multiple cohort and cross-sectional datasets. Each data source has independent sampling and complementary strengths (see Introduction), enabling more robust inferences than either data source in isolation. However, despite the large sample sizes used, we were potentially underpowered to robustly test for differences in the BMI and blood pressure association across time by age.

While our analysis accounted for the potential confounding role of socioeconomic factors, we cannot exclude the possibility of other unmeasured confounders. Many plausible confounders—such as preceding ill health and behavioural factors which influence both BMI and blood pressure—are likely to be partly but imperfectly captured by preceding socioeconomic position. We also accounted for missing data using multiple imputation and analytical weights, yet similarly cannot exclude the possibility that unmeasured factors associated with non-response may have biased our findings. The introduction of analytic weights in the HSE series in 2003 means that comparison of trends in association before and after this year should be treated with caution. While it is challenging to compare trends when blood pressure measurement devices have changed, we used regression equations based on calibration studies to account for this (Stang et al., 2006; Falaschetti et al., 2009). Interpretation of trends across time during these periods is thus dependent on the plausibility of the assumptions inherent in such calibration. We also accounted for antihypertensive medication by adding constant values to the raw SBP/DBP values for those on treatment. While the contribution of treatment use to our findings of change across time in association was estimated to be minimal, our approximation of its effect on BMI-blood pressure associations is imperfect owing to a lack of data on treatment, dose, and adherence (Tanamas et al., 2017).

## 6 Conclusion

The consequences of BMI may differ by both time period and age group—associations between BMI and SBP appear to have weakened in recent decades, particularly at older ages. If causal, the weakening strength of association may offset the impact of increases in obesity prevalence at the population level. However, BMI remains positively associated with SBP in all age groups, highlighting the future adverse consequences of the ongoing obesity epidemic. Finally, our results highlight the utility of using multiple datasets to obtain robust inferences regarding trends in risk factor-outcome associations across time; and the potential limitations of drawing inferences on long-term trends when comparing only two time-points.

## Funding

DB is supported by the 10.13039/501100000269Economic and Social Research Council (grant number ES/M001660/1), The Academy of Medical Sciences/Wellcome Trust (“Springboard Health of the Public in 2040” award: HOP001/1025), and 10.13039/501100000265Medical Research Council (MR/V002147/1). The HSE is funded by NHS Digital; SS is funded to conduct the annual 10.13039/501100000869HSE. RH and DON are respectively the Director and Harmonisation Theme Lead at CLOSER, which is funded by the 10.13039/501100000269Economic and Social Research Council (award reference: ES/K000357/1). The funders had no role in study design, data collection and analysis, decision to publish, or preparation of the manuscript.

## Declaration of Competing Interest

None.

## Appendix A Supplementary data

Supplementary material Image 1
