# Supplementary material for: Changes in the body mass index and blood pressure association across time: Evidence from multiple cross-sectional and cohort studies
Source: Prev Med. 2021 Dec;153:106825. doi: 10.1016/j.ypmed.2021.106825 (PMC8633761; doi:10.1016/j.ypmed.2021.106825)
Supplement: Supplementary file 1 — Supplementary material [file mmc1.docx]

**Supplementary materials for:**

**Bann et al. Changes in the body mass index and blood pressure association across time: Evidence from multiple cross-sectional and cohort studies**

Table of contents

[Participant selection flowchart: Birth cohort data 2](#_Toc82432302)

[Participant selection flowchart: Repeated cross-sectional data 3](#_Toc82432303)

[Observed data distributions 4](#_Toc82432304)

[Analyses showing sex-adjusted associations before and after accounting for treatment and socioeconomic factors 6](#_Toc82432305)

[Cross-sectional data restricted to 40-49 years age 7](#_Toc82432306)

[Test of change across time in the BMI-SBP association (interaction terms for BMI*year in HSE) 8](#_Toc82432307)

[Cohort data confined to English residents only 9](#_Toc82432308)

[Quantile regression results by year 10](#_Toc82432309)

[Diastolic blood pressure 11](#_Toc82432310)

[Sex-stratified (Birth cohort data): Systolic blood pressure 12](#_Toc82432311)

[Sex-stratified (Repeated cross-sectional data, ≥25 years): Systolic blood pressure 13](#_Toc82432312)

[Sex-stratified (Birth cohort data): Diastolic blood pressure 15](#_Toc82432313)

[Sex-stratified (Repeated cross-sectional data, ≥25 years): Diastolic blood pressure 16](#_Toc82432314)

## Participant selection flowchart: Birth cohort data


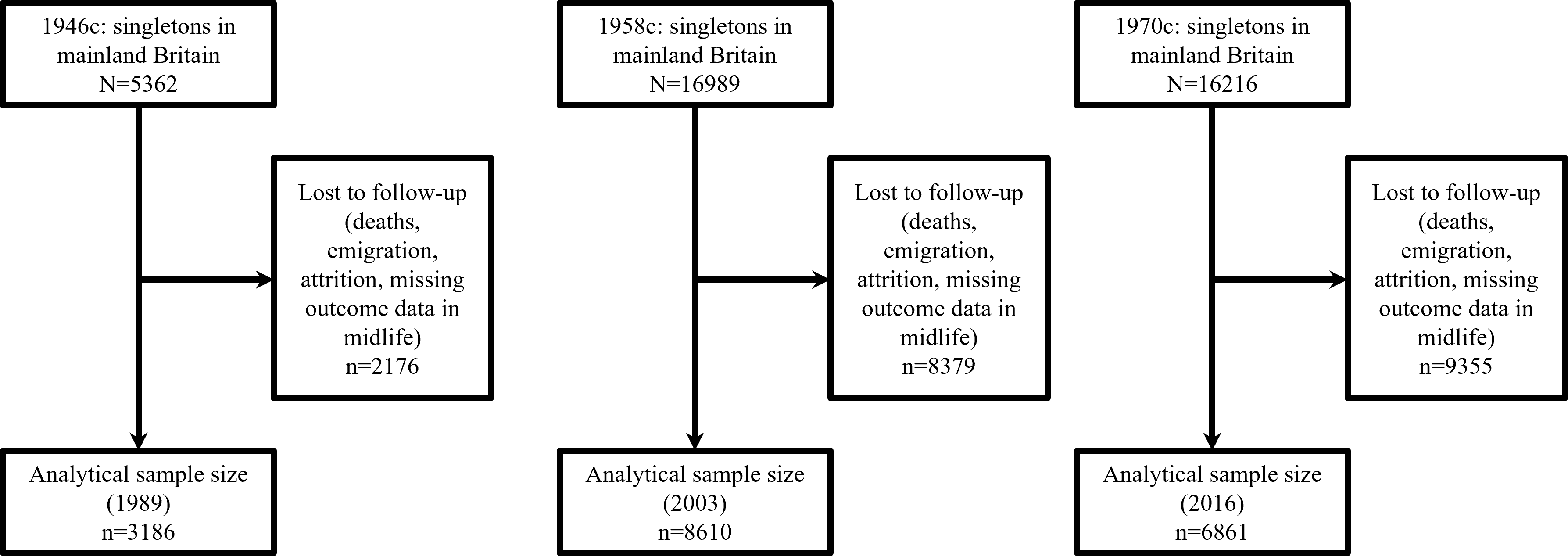


**Supplementary Figure 1. Participant selection flowchart for birth cohort data.**

## Participant selection flowchart: Repeated cross-sectional data

| **Participating households (response rate)^a^:** |  | **Interviewed ^b^:** |  | **Nurse visit ^b^:** |  | **Valid blood pressure and medicine data ^b,c^:** |  | **Complete education data  ^b,d^:** |  | **Valid BMI (analytical sample)** |
| --- | --- | --- | --- | --- | --- | --- | --- | --- | --- | --- |
| **1994:** N=9068 (77%) | **⟶** | N=13 757 | **⟶** | N=11 999 | **⟶** | N=11 653 | **⟶** | N=11 427 | **⟶** | N=11 025 |
| **1995:** N=9084 (78%) | **⟶** | N=14 040 | **⟶** | N=12 149 | **⟶** | N=11 044 | **⟶** | N= 10 427 | **⟶** | N= 9844 |
| **1996:** N=9350 (79%) | **⟶** | N=14 834 | **⟶** | N=12 641 | **⟶** | N=11 532 | **⟶** | N=10 900 | **⟶** | N=10 453 |
| **1997:** N=4905 (76%) | **⟶** | N=7530 | **⟶** | N=6717 | **⟶** | N=6066 | **⟶** | N=5764 | **⟶** | N=5492 |
| **1998:** N=9208 (74%) | **⟶** | N=14 027 | **⟶** | N=12 052 | **⟶** | N=10 597 | **⟶** | N=10 040 | **⟶** | N=9376 |
| **2000:** N=4788 (75%) | **⟶** | N=7134 | **⟶** | N=5989 | **⟶** | N=5070 | **⟶** | N=4847 | **⟶** | N=4421 |
| **2001:** N=9373 (74%) | **⟶** | N=13 873 | **⟶** | N=11 082 | **⟶** | N=9554 | **⟶** | N=9061 | **⟶** | N=8353 |
| **2002:** N=5112 (76%) | **⟶** | N=6436 | **⟶** | N=5217 | **⟶** | N=4287 | **⟶** | N=4051 | **⟶** | N=3736 |
| **2003:** N=8867 (73%) | **⟶** | N=13 200 | **⟶** | N=10 276 | **⟶** | N=8334 | **⟶** | N=7910 | **⟶** | N=7318 |
| **2005:** N=4546 (71%) | **⟶** | N=6728 | **⟶** | N=4897 | **⟶** | N=3907 | **⟶** | N=3822 | **⟶** | N=3449 |
| **2006:** N=8615 (68%) | **⟶** | N=12 698 | **⟶** | N=9522 | **⟶** | N=8016 | **⟶** | N=7836 | **⟶** | N=7134 |
| **2007:** N=4200 (66%) | **⟶** | N=6137 | **⟶** | N=4503 | **⟶** | N=3775 | **⟶** | N=3691 | **⟶** | N=3384 |
| **2008:** N=9191 (64%) | **⟶** | N=13 405 | **⟶** | N=9652 | **⟶** | N=8117 | **⟶** | N=7944 | **⟶** | N=7225 |
| **2009:** N=2832 (68%) | **⟶** | N=4137 | **⟶** | N=2950 | **⟶** | N=2526 | **⟶** | N=2467 | **⟶** | N=2265 |
| **2010:** N=5249 (66%) | **⟶** | N=7565 | **⟶** | N=5091 | **⟶** | N=3950 | **⟶** | N=3853 | **⟶** | N=3504 |
| **2011:** N=5338 (66%) | **⟶** | N=7755 | **⟶** | N=5225 | **⟶** | N=4042 | **⟶** | N=3979 | **⟶** | N=3573 |
| **2012:** N=5219 (64%) | **⟶** | N=7445 | **⟶** | N=4989 | **⟶** | N=4131 | **⟶** | N=4044 | **⟶** | N=3662 |
| **2013:** N=5416 (64%) | **⟶** | N=7926 | **⟶** | N=5673 | **⟶** | N=4813 | **⟶** | N=4716 | **⟶** | N=4280 |
| **2014:** N=5051 (62%) | **⟶** | N=7297 | **⟶** | N=5059 | **⟶** | N=4312 | **⟶** | N=4244 | **⟶** | N=3906 |
| **2015:** N=5111 (60%) | **⟶** | N=7325 | **⟶** | N=4977 | **⟶** | N=4191 | **⟶** | N=4114 | **⟶** | N=3756 |
| **2016:** N=5096 (59%) | **⟶** | N=7282 | **⟶** | N=4692 | **⟶** | N=4049 | **⟶** | N=3981 | **⟶** | N=3560 |
| **2017:** N=5137 (60%) | **⟶** | N=7350 | **⟶** | N=4889 | **⟶** | N=4135 | **⟶** | N=4076 | **⟶** | N=3654 |
| **2018:** N=5129 (59%) | **⟶** | N=7430 | **⟶** | N=4488 | **⟶** | N=3792 | **⟶** | N=3748 | **⟶** | N=3372 |
| **1994-2018** |  | **N=215,311** |  | **N=164,729** |  | **N=141,893** |  | **N=136,942** |  | **N=126,742** |

**Supplementary Figure 2. Participant selection flowchart for repeated cross-sectional data.**

**Notes: ^a^**All co-operating households in HSE (i.e. at least one person interviewed): response rate calculated as co-operating households / eligible households (full details are available in HSE documentation). BP data not available in 1999 or 2004 due to the oversampling of minority ethnic groups. **^b^**Adults aged 25 years and over. ^c^Participants who had exercised, eaten, drunk alcohol or smoked in the 30 minutes before BP measurements were excluded from BP data. ^d^Education data excludes those with ‘other’ qualifications.

## Observed data distributions

1. Cohort studies


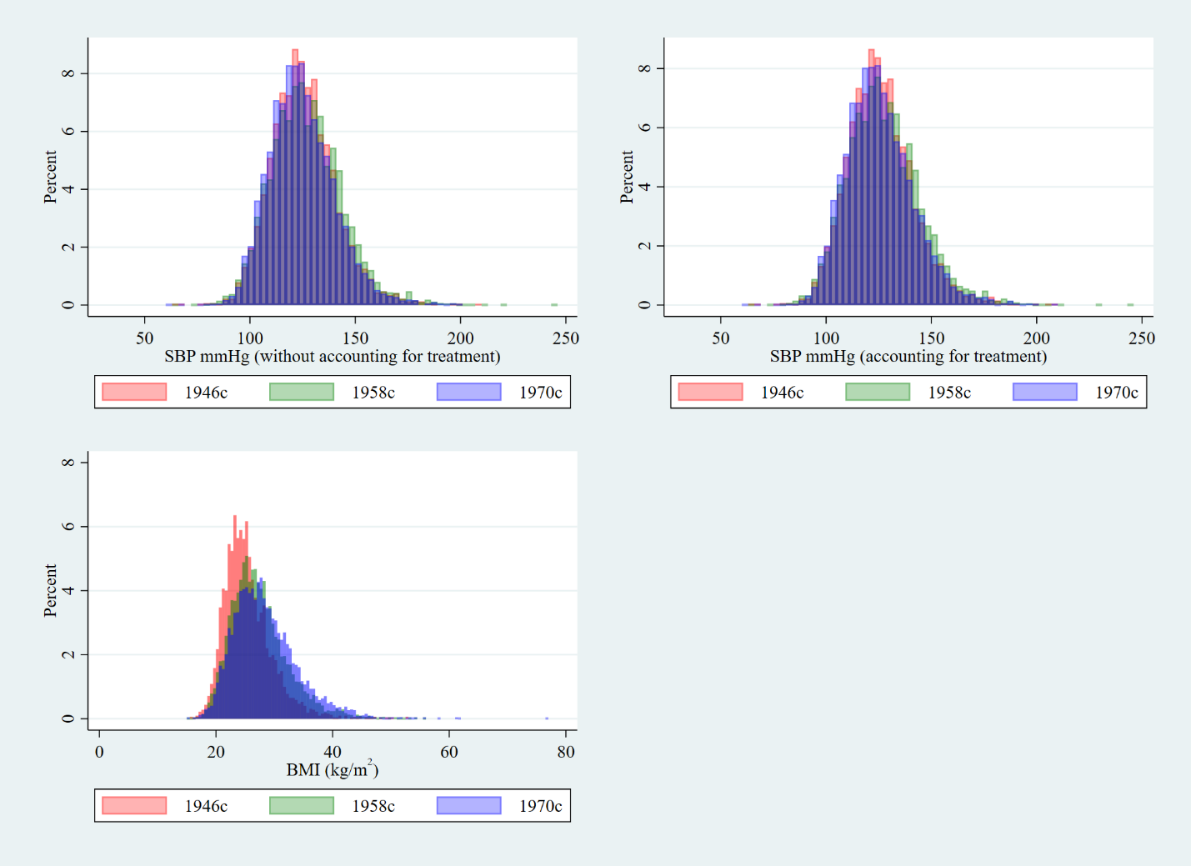


1. Cross-sectional studies:


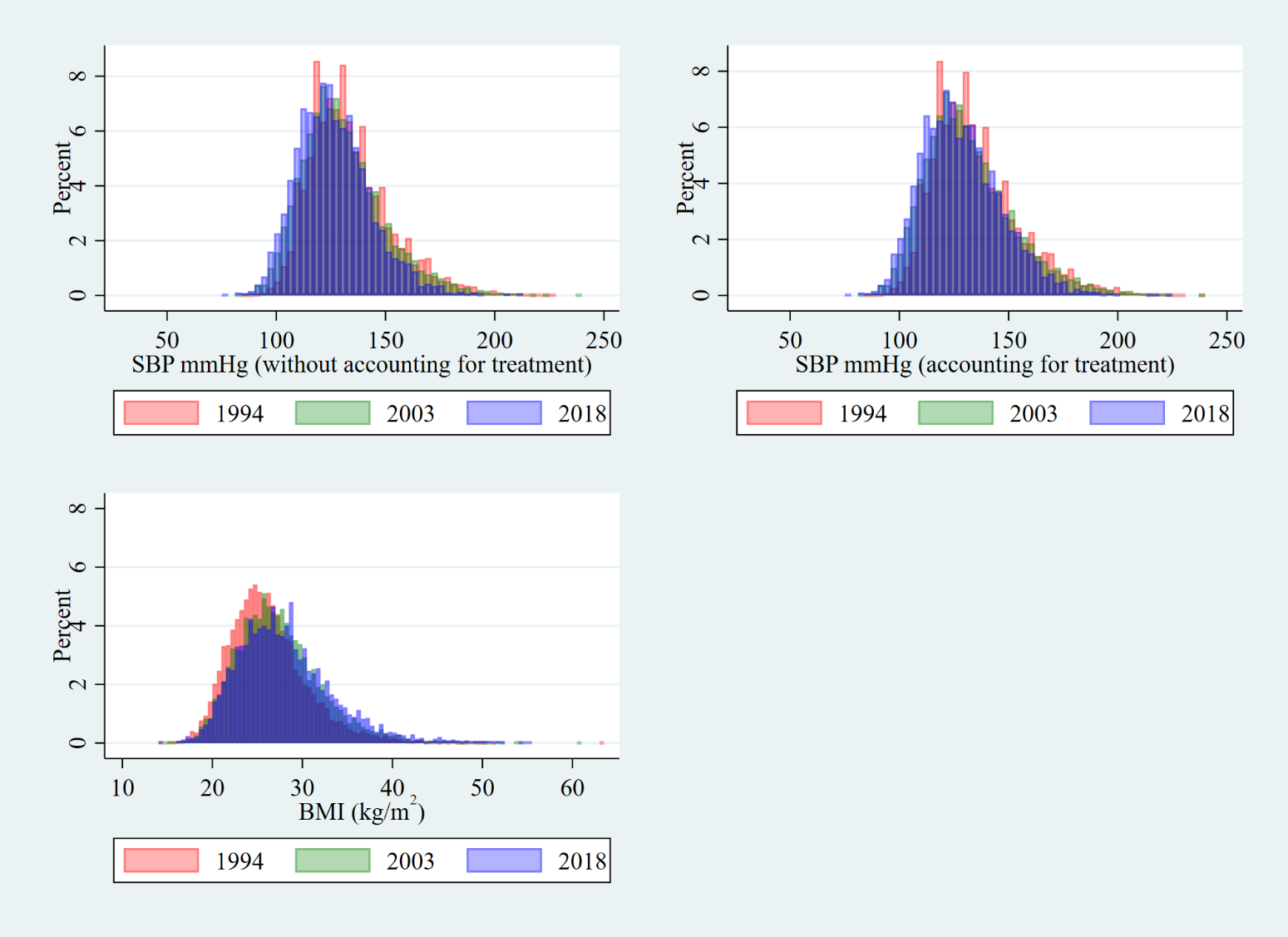


**Supplementary Figure 3. Histograms of SBP (mmHg, before (raw) and after accounting for antihypertensive treatment) and observed BMI in i) 1946, 1958 and 1970 birth cohorts (18,402 for SBP; N=18,658 for BMI) and ii) repeated cross-sectional data in 1994, 2003, and 2018 (N=136,942 for SBP; N=126,742 for BMI).**

## Analyses showing sex-adjusted associations before and after accounting for treatment and socioeconomic factors


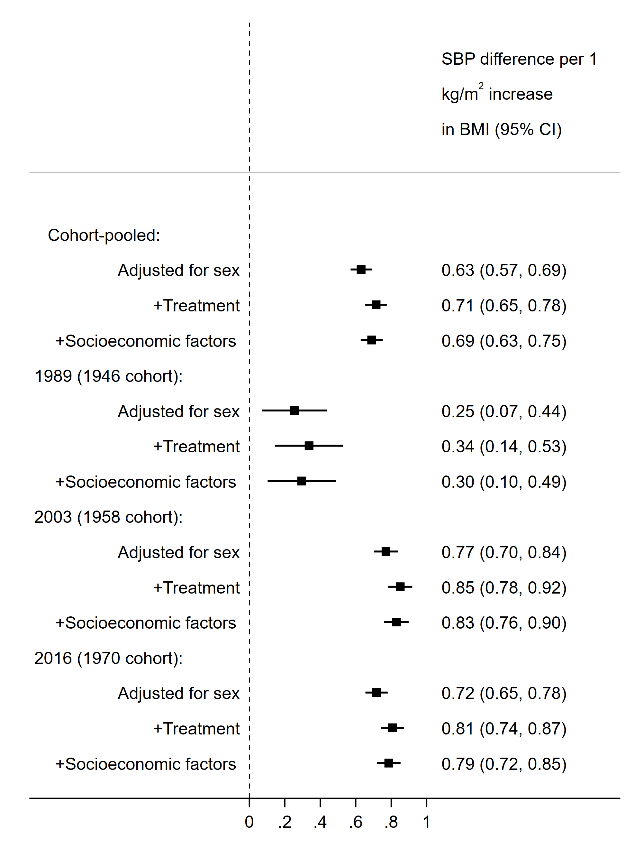

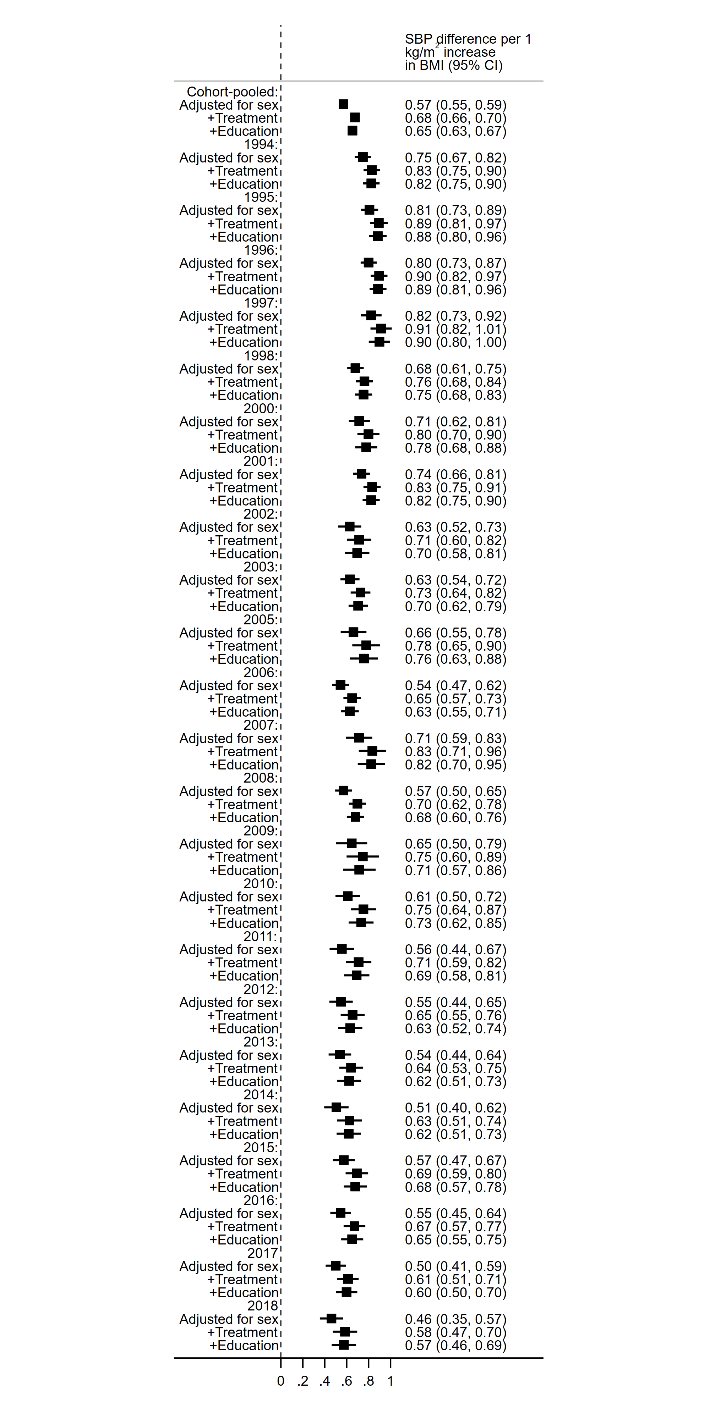


**Supplementary Figure 4. Associations between body mass index (kg/m^2^) and systolic blood pressure (mmHg) in midlife (42-46 years, from birth cohort data, left panel) and across adulthood (≥25 years, from repeated cross-sectional data, right panel).**

Note: Adjusted for sex: model estimated on raw SBP data; +Treatment: SBP accounted for treatment use by adding a constant of 10mmHg for those using antihypertensive medication. Cohort studies: socioeconomic factors comprise mother’s education and cohort member’s own education, social class at birth, and midlife social class. Sample size for cohorts N=18,657, HSE=126,742; sample sizes in each cohort / survey year shown in Supplementary Figures 1 and 2, respectively.

## Cross-sectional data restricted to 40-49 years age


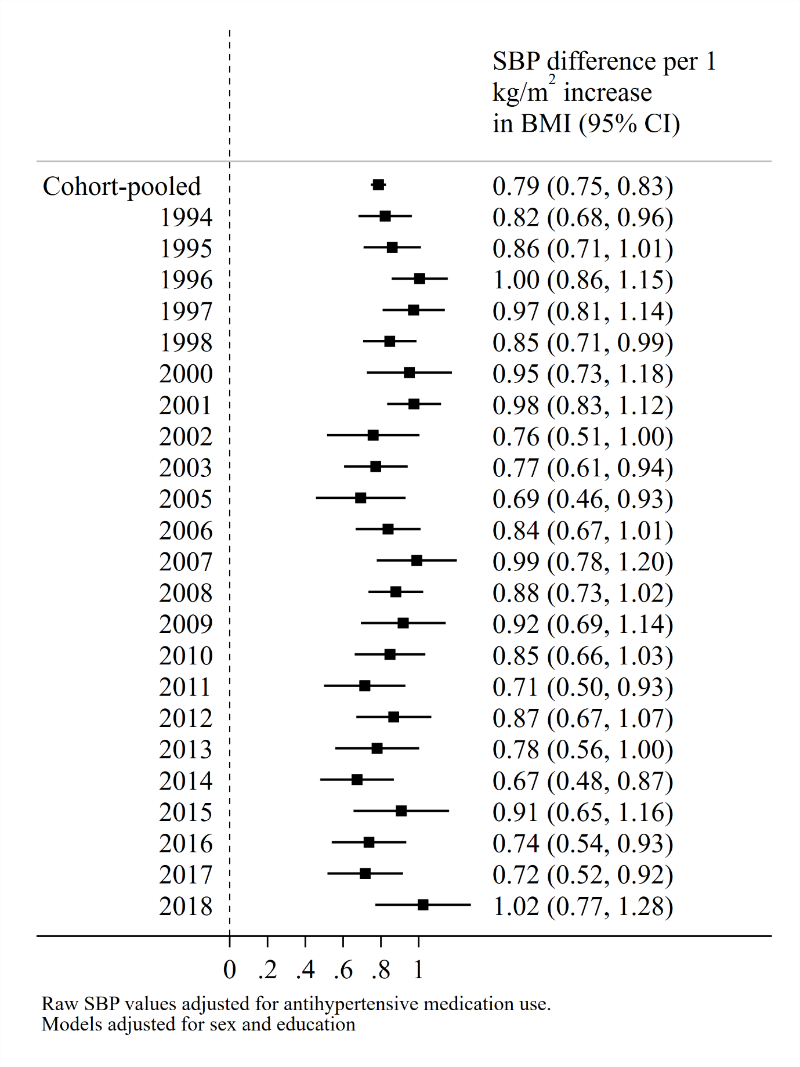


**Supplementary Figure 5. Associations between body mass index (kg/m^2^) and blood pressure (mmHg) in midlife (from independent, repeated cross-sectional data).**

Note: BP adjusted for treatment by adding a constant of 10mmHg to SBP for those using antihypertensive medication. Sample size for HSE=126,742; sample sizes in each survey year shown in Supplementary Figure 2.

## Test of change across time in the BMI-SBP association (interaction terms for BMI*year in HSE)

**Supplementary Table 1.**  Test of change across time in the BMI-SBP association in the Health Survey for England, by age group and year**.**

| **Slope for BMI*Year** |  | **95% CI** | |  |  |
| --- | --- | --- | --- | --- | --- |
|  | **Estimate** | **Lower** | **Upper** | **P-value** | **N** |
| **25+:** |  |  |  |  |  |
| All years | -0.011 | -0.014 | -0.008 | <0.001 | 126,742 |
| 1994-2002 | -0.016 | -0.028 | -0.005 | 0.004 | 62,700 |
| 2003-2018 | -0.009 | -0.015 | -0.004 | 0.001 | 64,042 |
|  |  |  |  |  |  |
| **25-54:** |  |  |  |  |  |
| All years | -0.006 | -0.009 | -0.003 | <0.001 | 73,750 |
| 1994-2002 | -0.010 | -0.021 | 0.002 | 0.106 | 39,267 |
| 2003-2018 | -0.005 | -0.011 | 0.002 | 0.177 | 34,483 |
|  |  |  |  |  |  |
| **55+:** |  |  |  |  |  |
| All years | -0.010 | -0.015 | -0.005 | <0.001 | 52,992 |
| 1994-2002 | -0.013 | -0.036 | 0.010 | 0.27 | 23,433 |
| 2003-2018 | -0.010 | -0.020 | 0.001 | 0.086 | 29,559 |
|  |  |  |  |  |  |
| **40-49:** |  |  |  |  |  |
| All years | -0.002 | -0.007 | 0.003 | 0.333 | 25,941 |
| 1994-2002 | 0.004 | -0.018 | 0.026 | 0.736 | 12,881 |
| 2003-2018 | -0.002 | -0.014 | 0.010 | 0.749 | 13,060 |

Notes: estimates obtained from separate linear regression models with SBP as the outcome (BP adjusted for treatment by adding a constant of 10mmHg to SBP for those using antihypertensive medication), and adjusting for sex and education attainment. A negative sign for BMI*year indicated a weakening BMI and SBP association in subsequent years.

## Cohort data confined to English residents only


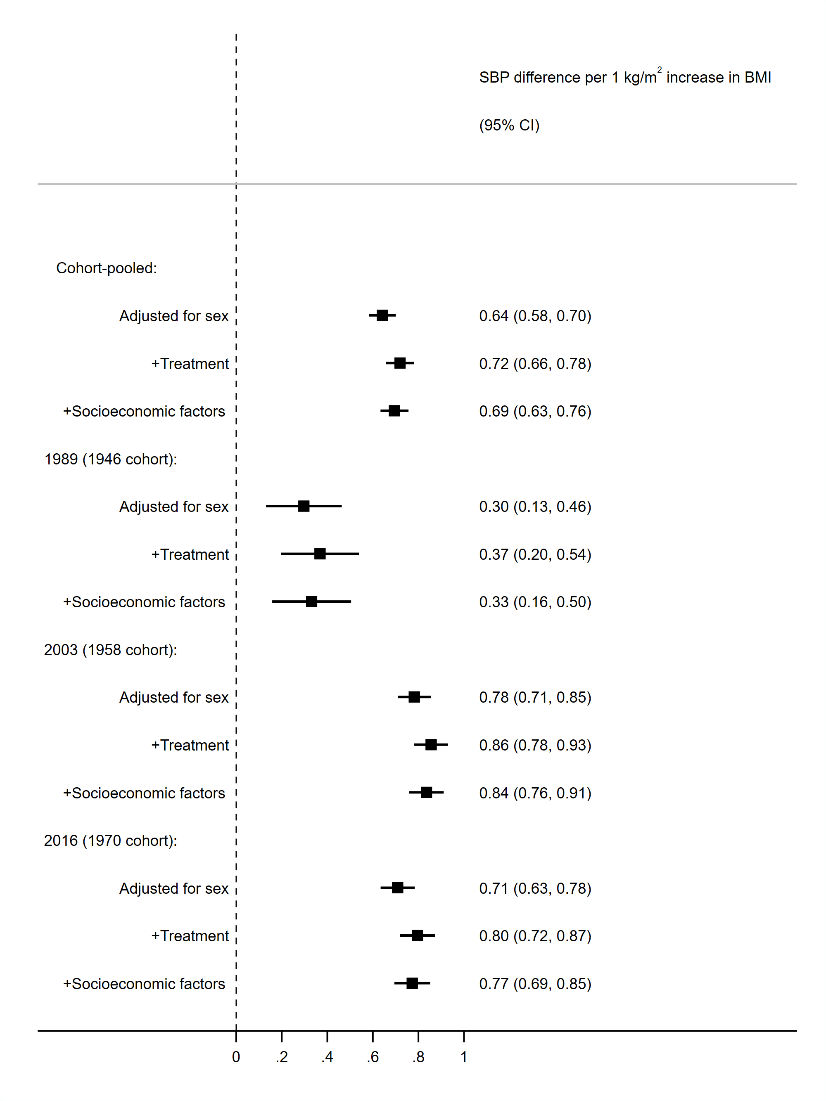


**Supplementary Figure 6. Associations between body mass index (kg/m^2^) and blood pressure (mmHg) in midlife (from cohort data confined to English based participants only).** Note: BP adjusted for treatment by adding a constant of 10mmHg to SBP for those using antihypertensive medication. Additional adjustment made for mother’s education and cohort member’s own education, social class at birth, and social class in midlife. Sample size for cohorts N=18,657; sample sizes in each cohort shown in Supplementary Figure 1.

## ***Quantile regression results by year***


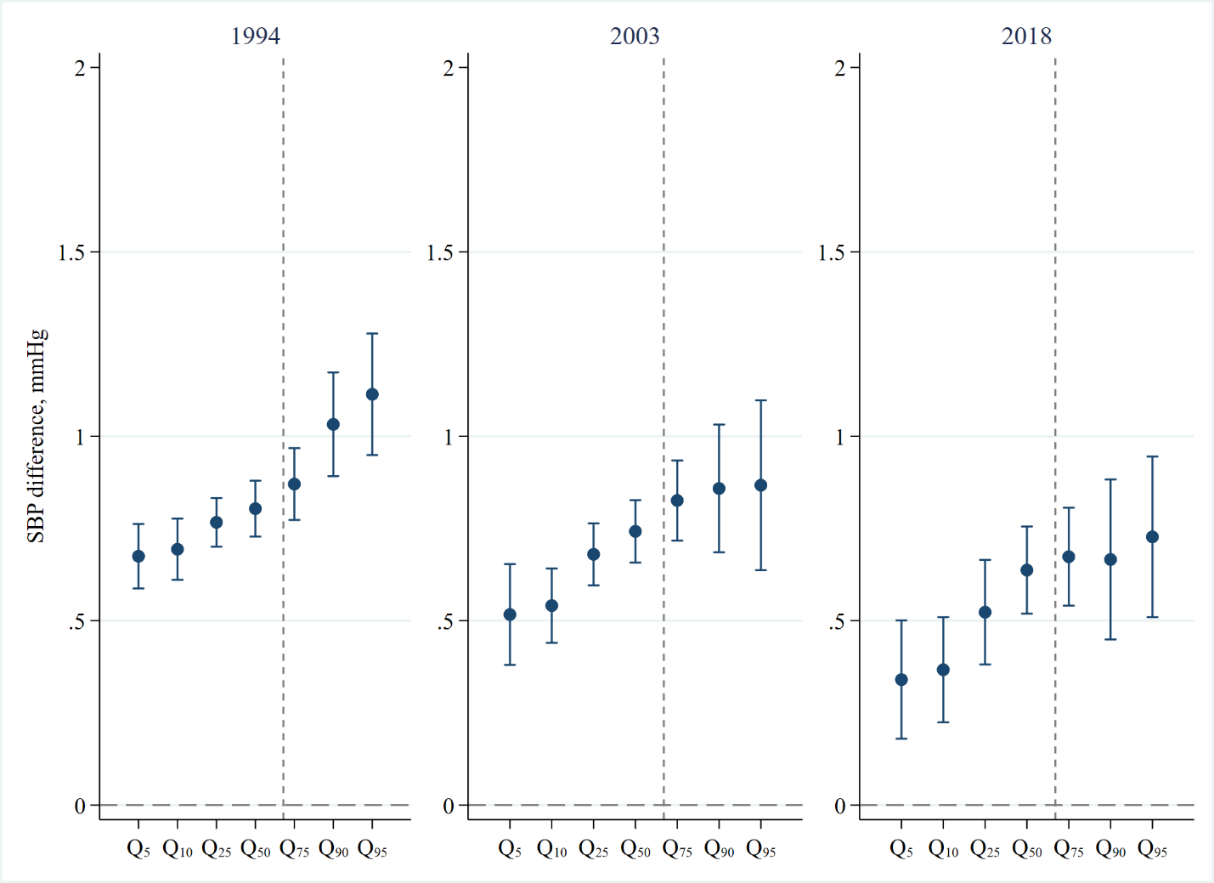


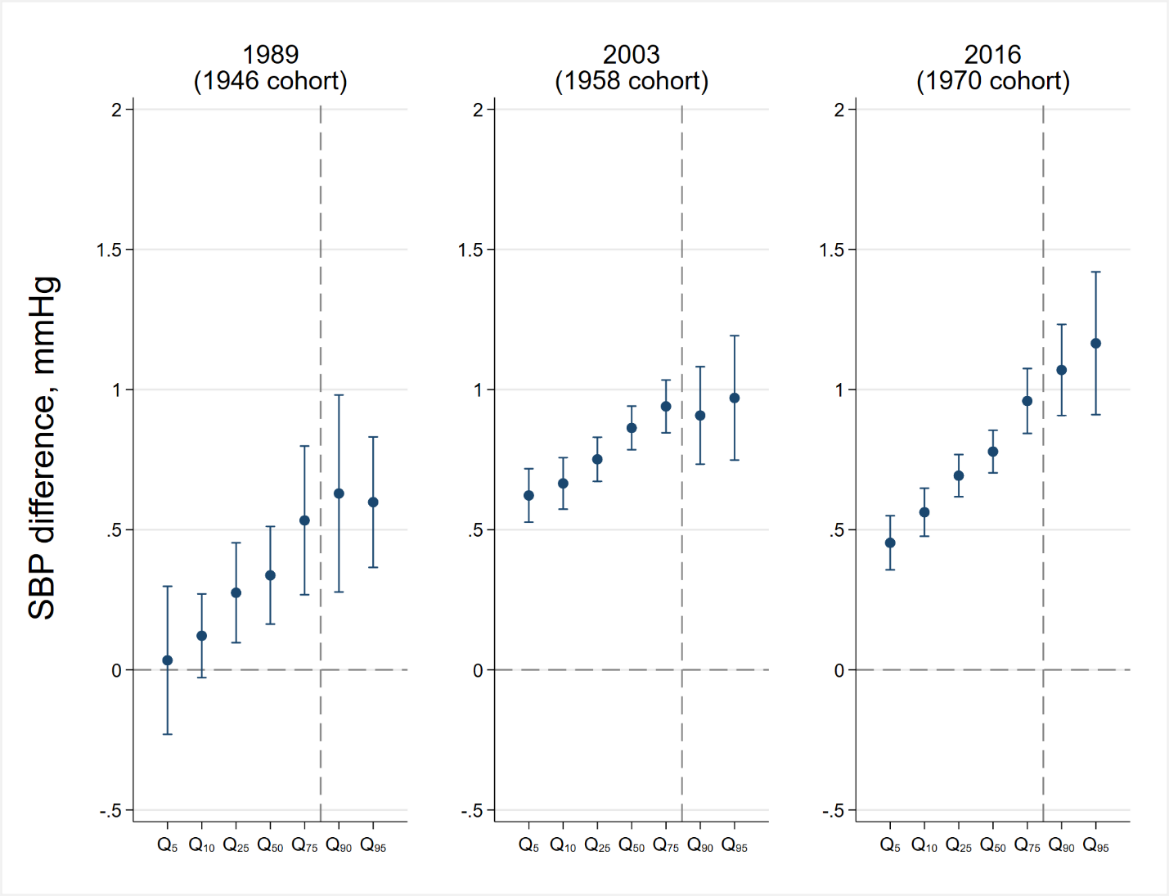


**Supplementary Figure 7. Associations between body mass index and systolic blood pressure quantiles (mmHg) across adulthood (≥25 years, from repeated cross-sectional data, top panel), and midlife (43-46 years, from birth cohort data, bottom panel).** Estimates show the difference in SBP per 1 unit increase in BMI at specified centiles of the SBP distribution; for example, Q50 shows the difference at the median. Note: raw SBP values adjusted for use of antihypertensive medication (by adding a constant of 10mmHg for those using antihypertensive medication); estimates are adjusted for sex and education attainment.

Diastolic blood pressure


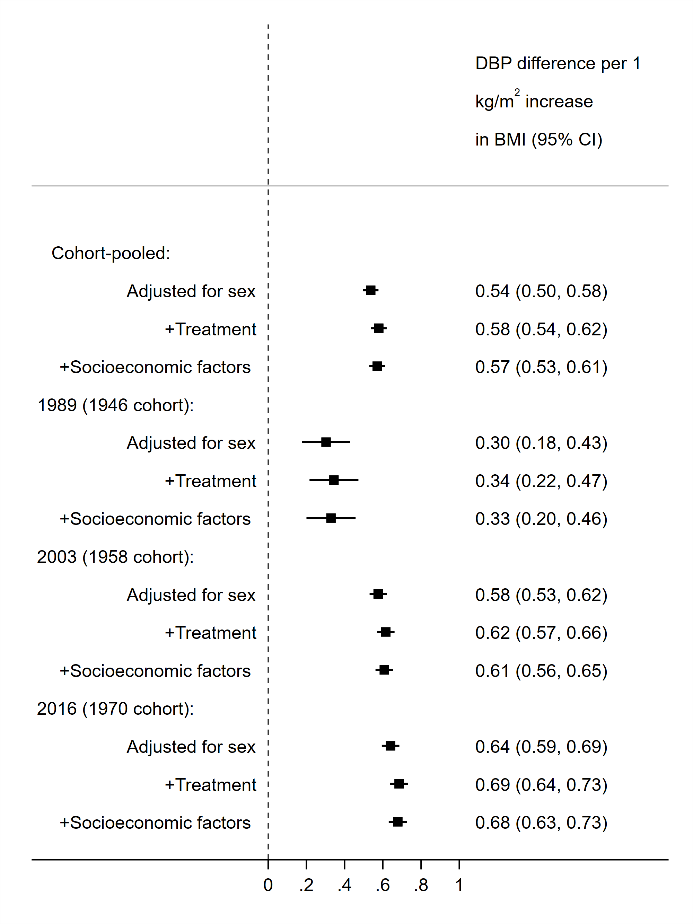

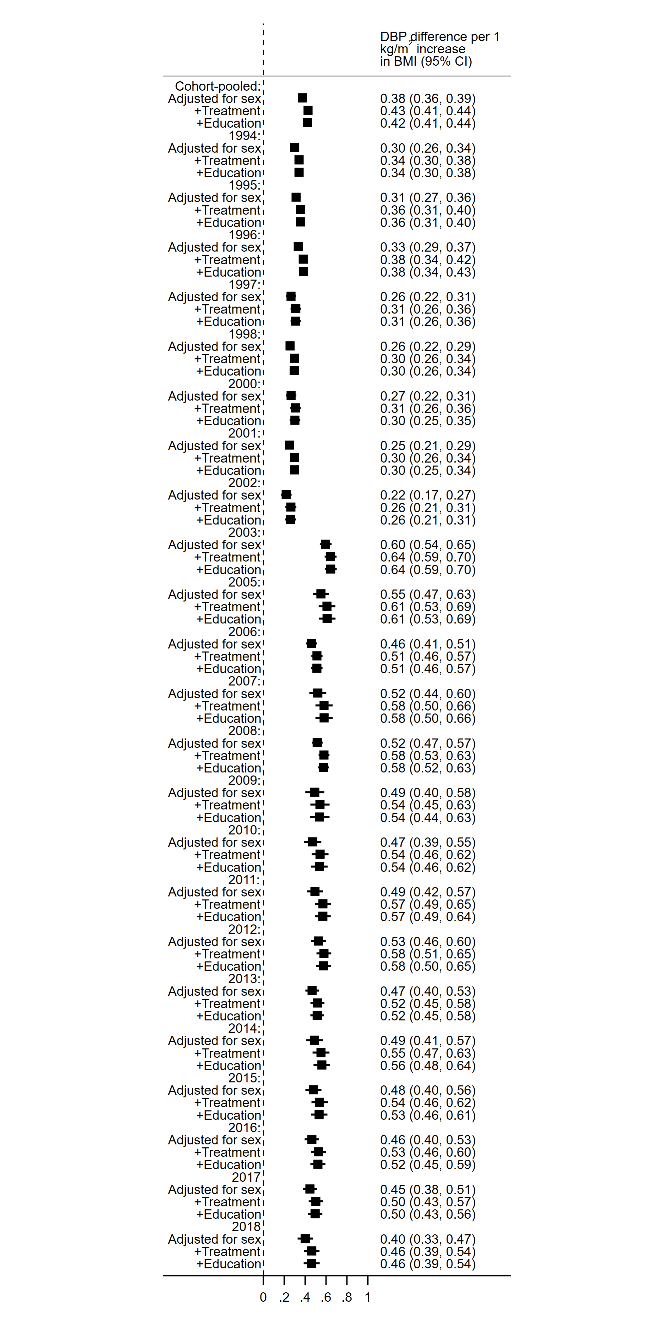


**Supplementary Figure 8. Associations between body mass index (kg/m^2^) and diastolic blood pressure (mmHg) in midlife (42-46 years, from birth cohort data, left panel) and across adulthood (≥25 years, from repeated cross-sectional data, right panel).**

Note: Adjusted for sex: model estimated on raw DBP data; +Treatment: DBP adjusted for treatment by adding a constant of 5mmHg for those using antihypertensive medication. Cohort studies: socioeconomic factors comprise mother’s education and cohort member’s own education, social class at birth, and midlife social class. Sample size for cohorts N=18,657, HSE=126,742; sample sizes in each cohort / survey year shown in Supplementary Figures 1 and 2, respectively.

## Sex-stratified (Birth cohort data): Systolic blood pressure


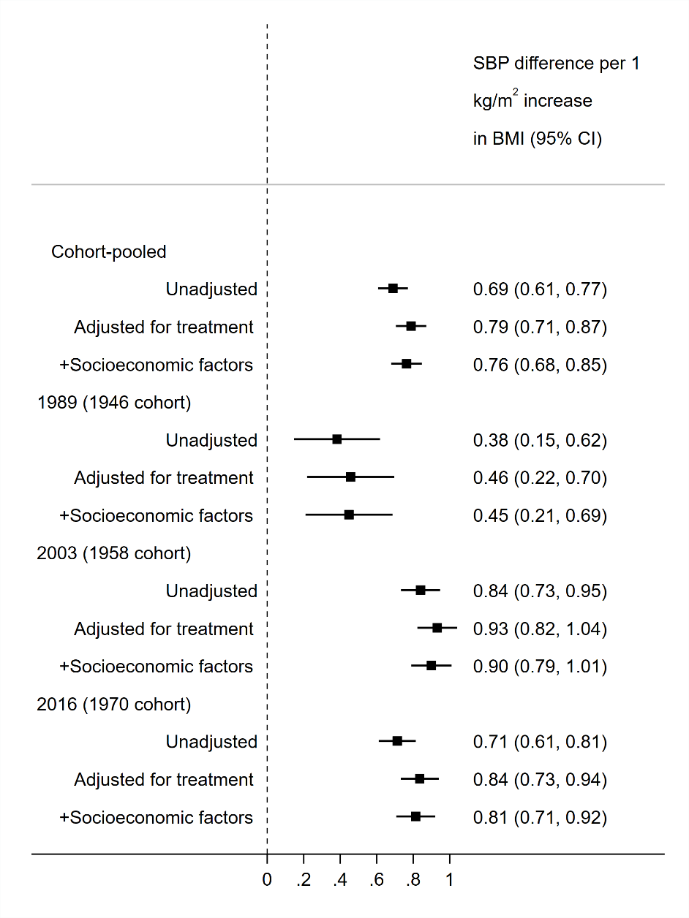

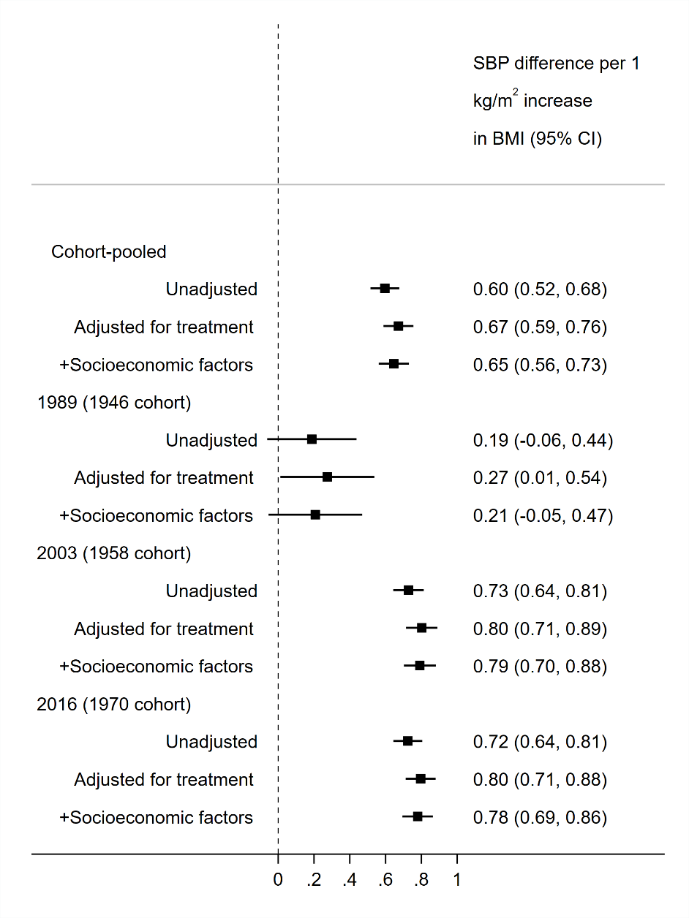


**Supplementary Figure 9A. Associations between body mass index (kg/m^2^) and systolic blood pressure (mmHg) during midlife (42-46 years, from birth cohort data) in males (left panel) and females (right panel).** Note: Unadjusted: model estimated on raw SBP data; BP adjusted for treatment by adding a constant of 10mmHg to SBP for those using antihypertensive medication. Socioeconomic factors comprise mother’s education and cohort member’s own education, social class at birth, and midlife social class.

Sample sizes for birth cohorts (men): cohort-pooled (N=9,190); 1946 cohort (N=1,596); 1958 cohort (N=4,296); 1970 cohort (N=3,298)

Sample sizes for birth cohorts (women): cohort-pooled (N=9,467); 1946 cohort (N=1,590); 1958 cohort (N=4,314); 1970 cohort (N=3,563)

## Sex-stratified (Repeated cross-sectional data, ≥25 years): Systolic blood pressure

…Supplementary Figure 9 continued.


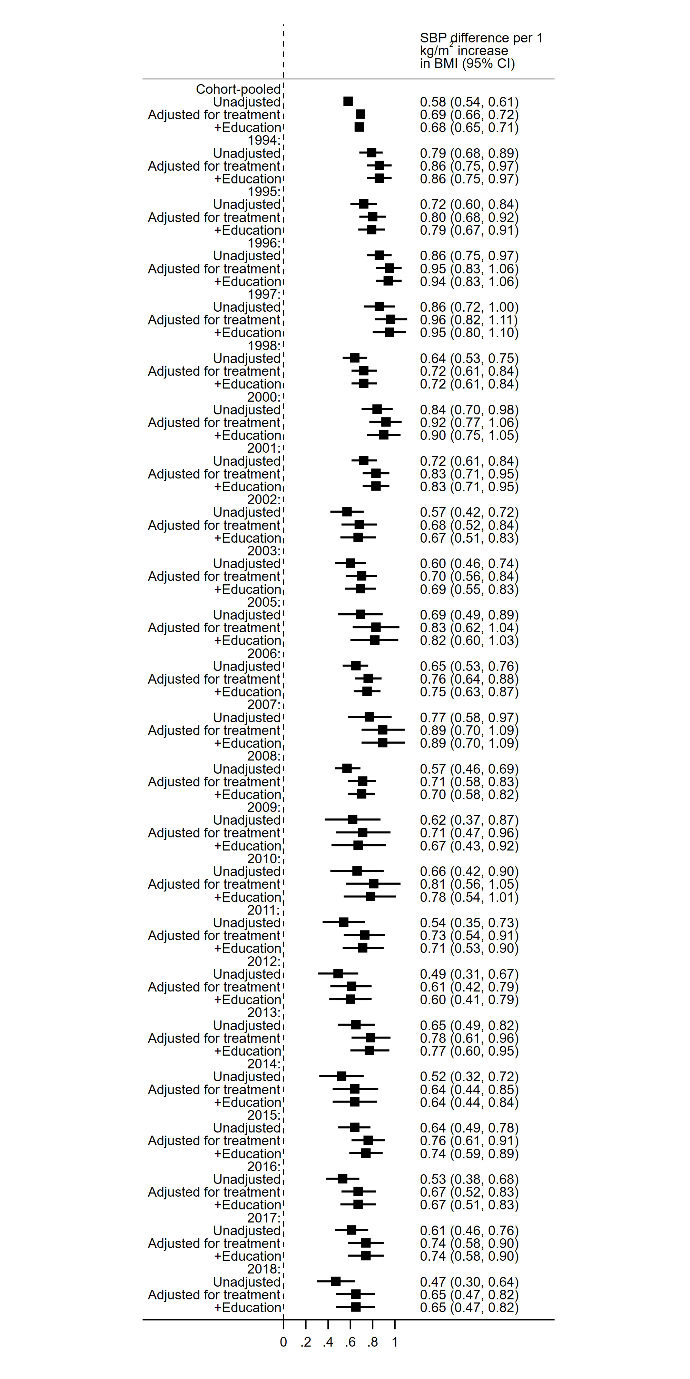

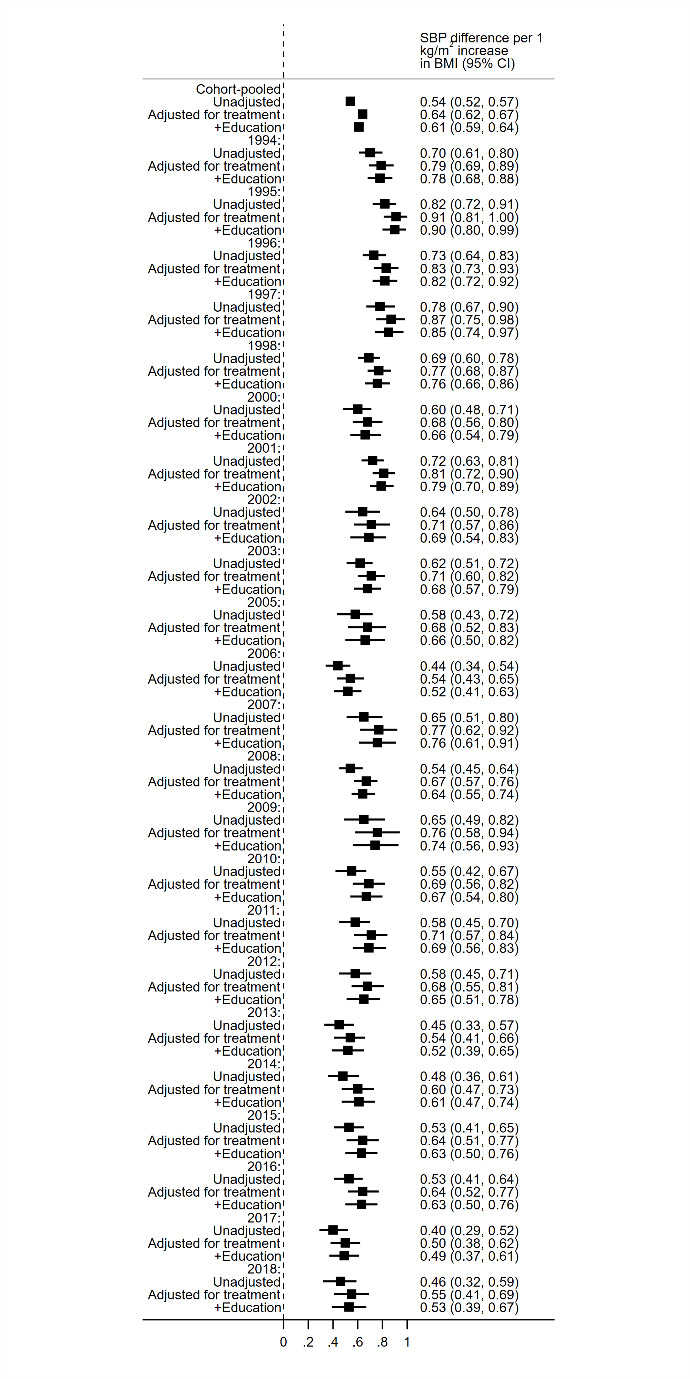


**Supplementary Figure 9B. Associations between body mass index (kg/m^2^) and systolic blood pressure (mmHg) across adulthood (≥25 years, from repeated cross-sectional data) in males (left panel) and females (right panel).** Note: Unadjusted: model estimated on raw SBP data; BP adjusted for treatment by adding a constant of 10mmHg to SBP for those using antihypertensive medication.

Sample sizes for HSE (men): cohort-pooled (N=58,322); 1994 (N=5102); 1995 (N=4679); 1996 (N=4896); 1997 (N=2599); 1998 (N=4362); 2000 (N=2054); 2001 (N=3912); 2002 (N=1710); 2003 (N=3408); 2005 (N=1561); 2006 (N=3294); 2007 (N=1565); 2008 (N=3312); 2009 (N=1037); 2010 (N=1531); 2011 (N=1561); 2012 (N=1649); 2013 (N=1943); 2014 (N=1739); 2015 (N=1717); 2016 (N=1587); 2017 (N=1603); 2018 (N=1501)

Sample sizes for HSE (women): cohort-pooled (N=68,420); 1994 (N=5923); 1995 (N=5165); 1996 (N=5557); 1997 (N=2893); 1998 (N=5014); 2000 (N=2367); 2001 (N=4441); 2002 (N=2026); 2003 (N=3910); 2005 (N=1888); 2006 (N=3840); 2007 (N=1819); 2008 (N=3913); 2009 (N=1228); 2010 (N=1973); 2011 (N=2012); 2012 (N=2013); 2013 (N=2337); 2014 (N=2167); 2015 (N=2039); 2016 (N=1973); 2017 (N=2051); 2018 (N=1871)

## Sex-stratified (Birth cohort data): Diastolic blood pressure


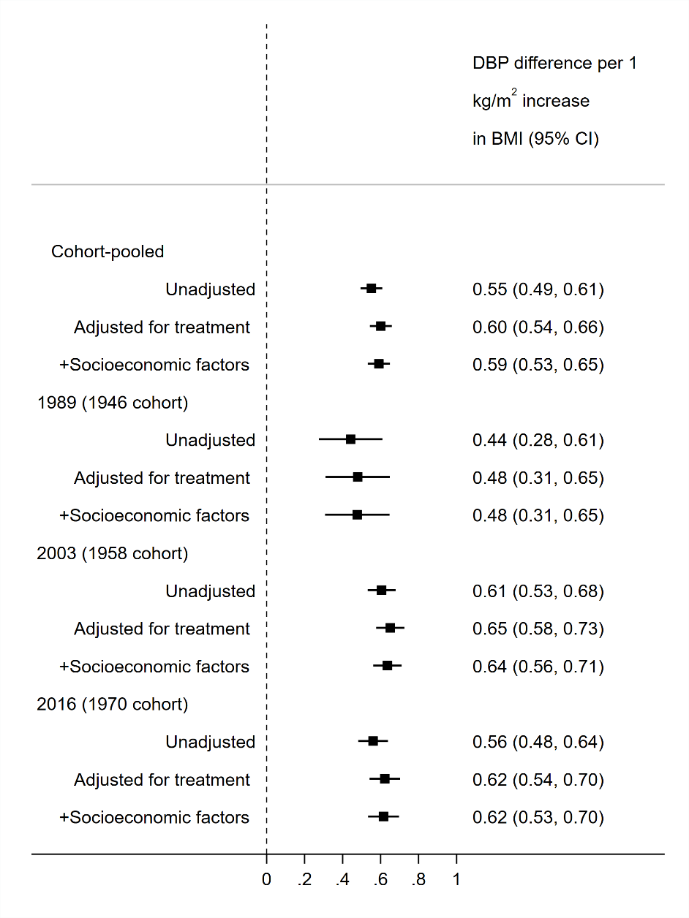

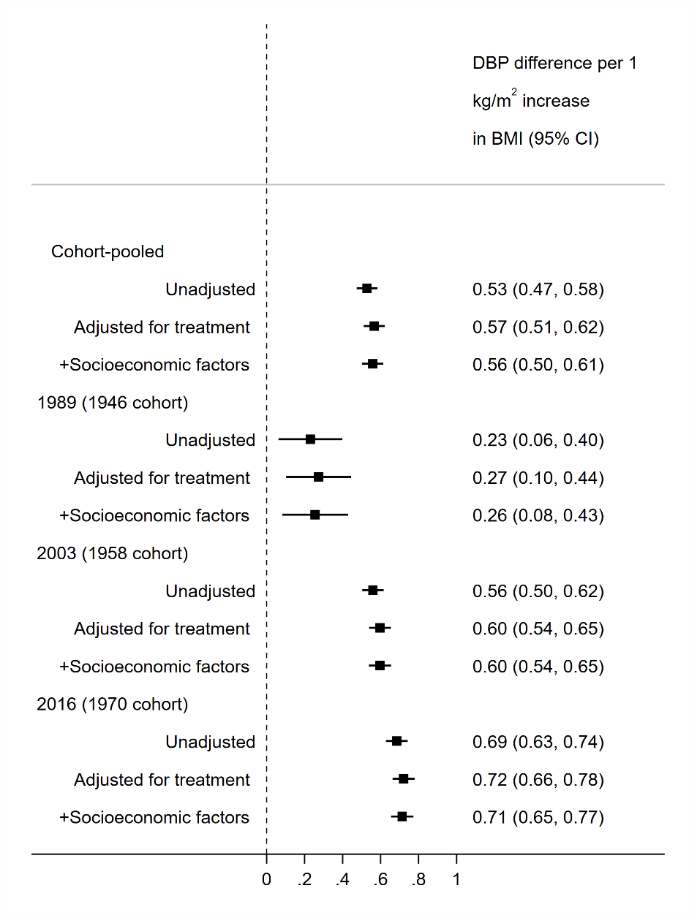


**Supplementary Figure 10A. Associations between body mass index (kg/m^2^) and diastolic blood pressure (mmHg) across adulthood (≥25 years, from birth cohort data) in males (left panel) and females (right panel).** Note: Unadjusted: model estimated on raw DBP data; BP adjusted for treatment by adding a constant of 5mmHg to DBP for those using antihypertensive medication.

Sample sizes for birth cohorts (men): cohort-pooled (N=9,190); 1946 cohort (N=1,596); 1958 cohort (N=4,296); 1970 cohort (N=3,298)

Sample sizes for birth cohorts (women): cohort-pooled (N=9,467); 1946 cohort (N=1,590); 1958 cohort (N=4,314); 1970 cohort (N=3,563)

## Sex-stratified (Repeated cross-sectional data, ≥25 years): Diastolic blood pressure

…Supplementary Figure 10 continued.


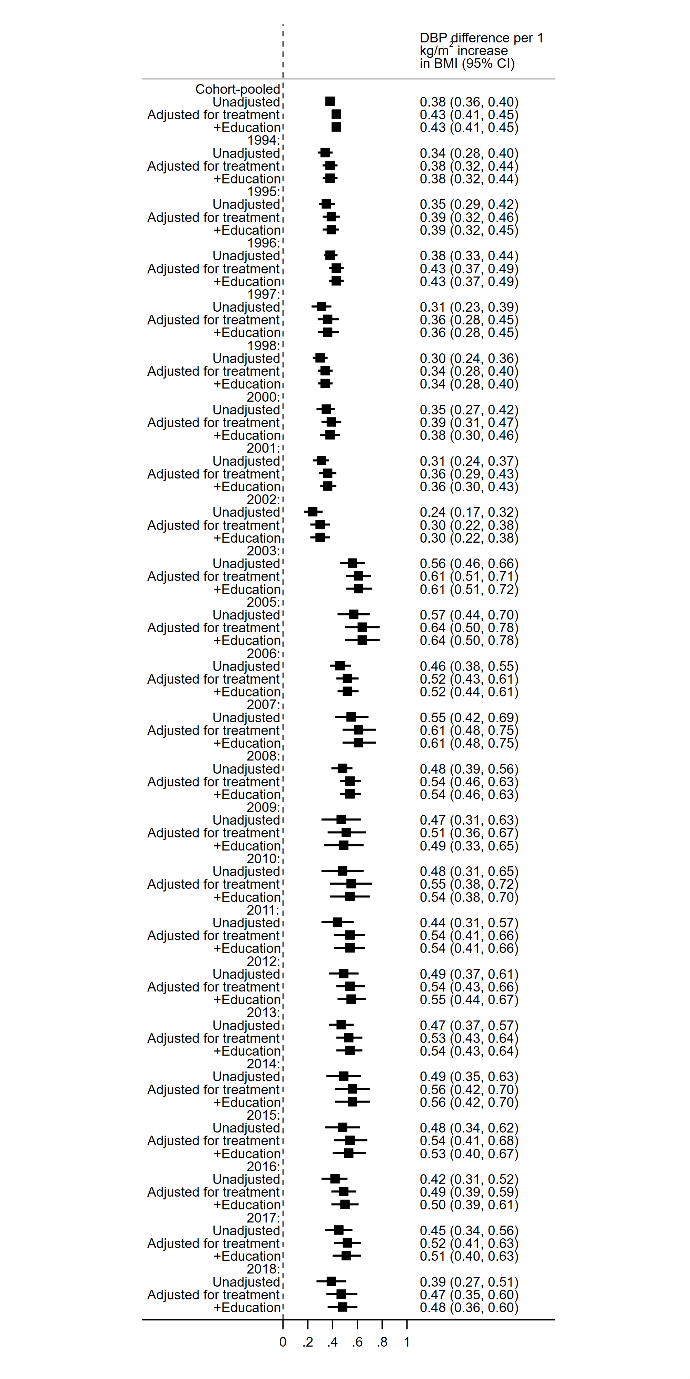

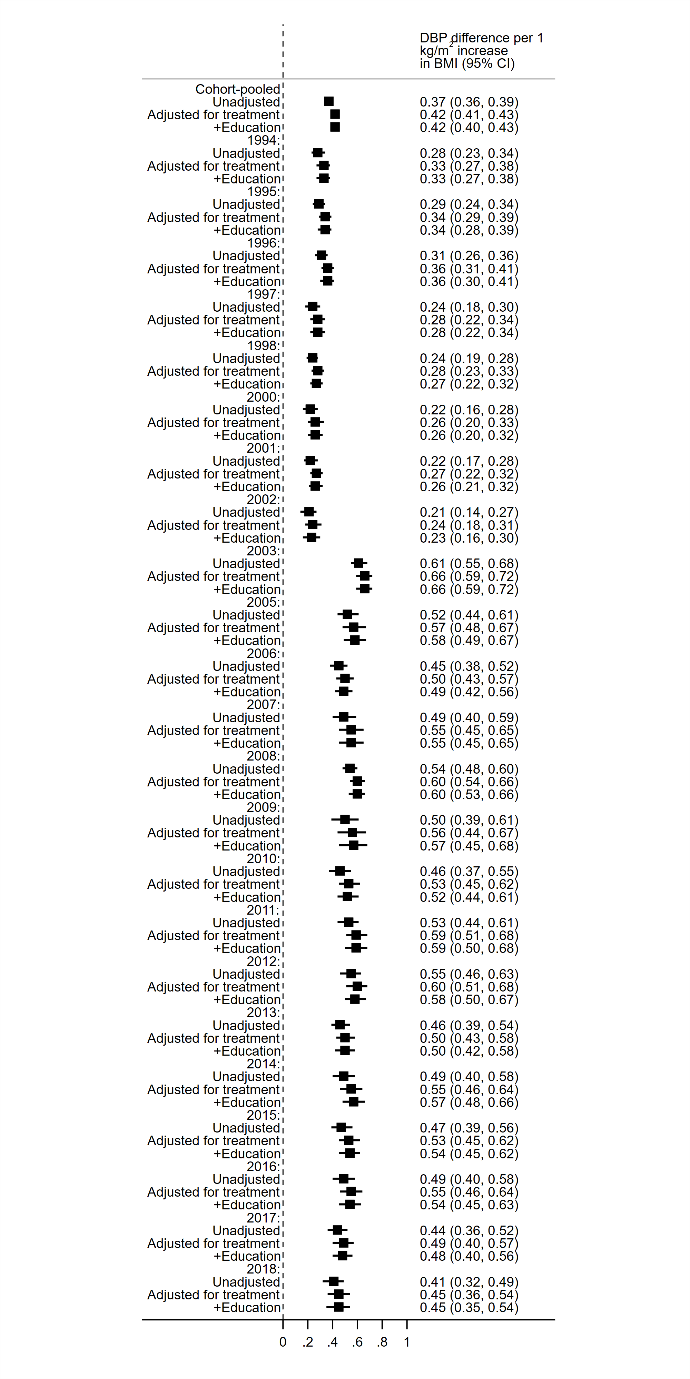


**Supplementary Figure 10B. Associations between body mass index (kg/m^2^) and diastolic blood pressure (mmHg) across adulthood (≥25 years, from repeated cross-sectional data) in males (left panel) and females (right panel).** Note: Unadjusted: model estimated on raw DBP data; BP adjusted for treatment by adding a constant of 5mmHg to DBP for those using antihypertensive medication.

Sample sizes for HSE (men): cohort-pooled (N=58,322); 1994 (N=5102); 1995 (N=4679); 1996 (N=4896); 1997 (N=2599); 1998 (N=4362); 2000 (N=2054); 2001 (N=3912); 2002 (N=1710); 2003 (N=3408); 2005 (N=1561); 2006 (N=3294); 2007 (N=1565); 2008 (N=3312); 2009 (N=1037); 2010 (N=1531); 2011 (N=1561); 2012 (N=1649); 2013 (N=1943); 2014 (N=1739); 2015 (N=1717); 2016 (N=1587); 2017 (N=1603); 2018 (N=1501)

Sample sizes for HSE (women): cohort-pooled (N=68,420); 1994 (N=5923); 1995 (N=5165); 1996 (N=5557); 1997 (N=2893); 1998 (N=5014); 2000 (N=2367); 2001 (N=4441); 2002 (N=2026); 2003 (N=3910); 2005 (N=1888); 2006 (N=3840); 2007 (N=1819); 2008 (N=3913); 2009 (N=1228); 2010 (N=1973); 2011 (N=2012); 2012 (N=2013); 2013 (N=2337); 2014 (N=2167); 2015 (N=2039); 2016 (N=1973); 2017 (N=2051); 2018 (N=1871)
